# IGFBP7 inhibits cell proliferation by suppressing AKT activity and cell cycle progression in thyroid carcinoma

**DOI:** 10.1186/s13578-019-0310-2

**Published:** 2019-06-06

**Authors:** Le Zhang, Rong Lian, Jingjing Zhao, Xianming Feng, Runyi Ye, Lingxiao Pan, Jueheng Wu, Mengfeng Li, Yongbo Huan, Junchao Cai

**Affiliations:** 1 Key Laboratory of Tropical Disease Control, Ministry of Education, Sun Yat-sen University, 74 Zhongshan Er Road, Guangzhou, 510080 Guangdong China; 2 Department of Microbiology, Zhongshan School of Medicine, Sun Yat-sen University, Guangzhou, 510080 Guangdong China; 3 Department of Cardiology, The First Affiliated Hospital, Sun Yat-sen University, Guangzhou, 510080 Guangdong China; 4 NHC Key Laboratory on Assisted Circulation of the First Affiliated Hospital, Sun Yat-sen University, Guangzhou, 510080 Guangdong China; 5 Department of Breast and Thyroid Surgery, The First Affiliated Hospital, Sun Yat-sen University, Guangzhou, 510080 Guangdong China; 6 Department of Breast Surgery, The First Affiliated Hospital of Guangzhou Medical University, Guangzhou, 510080 Guangdong China; 7 State Key Laboratory of Respiratory Diseases and Guangzhou Institute of Respiratory Diseases, The First Affiliated Hospital of Guangzhou Medical University, 151 Yanjiang Road, Guangzhou, 510000 Guangdong China

**Keywords:** IGFBP7, Thyroid cancer, Cell cycle, AKT, Proliferation

## Abstract

**Background:**

Thyroid cancer is the most common malignant endocrine tumor and is classified into papillary thyroid cancer (PTC), follicular thyroid cancer (FTC) and anaplastic thyroid cancer (ATC), which have substantially different characteristics. Insulin-like growth factor binding protein 7 (IGFBP7) has recently been recognized as a tumor suppressor in many cancer types. However, the expression pattern of IGFBP7 and its biological function in various types of thyroid carcinoma remain poorly understood.

**Results:**

We found that the protein levels of IGFBP7 in FTC and ATC tissues were significantly lower or even absent compared with those in normal thyroid, benign thyroid adenoma and classical PTC tissues. Moreover, overexpression of IGFBP7 in two undifferentiated ATC cell lines, ARO and FRO, and one differentiated FTC cell line, WRO, significantly inhibited cell proliferation in vitro. In vivo experiments revealed that ectopic IGFBP7 expression markedly suppressed growth of tumor xenografts derived from these thyroid cancer cell lines, while IGFBP7 silencing accelerated tumor growth. At the mechanistic level, overexpression of IGFBP7 dramatically suppressed phosphorylation-mediated activation and kinase activity of AKT, causing an upregulation of cyclin-dependent kinase (CDK) inhibitors p27^Kip1^ and p21^Cip1^ and induction of G1/S cell cycle arrest, while silencing IGFBP7 exerted the opposite effects.

**Conclusions:**

IGFBP7 expression is decreased or even absent in FTC and ATC. Acting as a cell cycle repressor, IGFBP7 plays an important tumor-suppressive role in human thyroid cancer, especially in FTC and ATC subtypes and may represent a promising biomarker and therapeutic target for human thyroid cancer treatment.

**Electronic supplementary material:**

The online version of this article (10.1186/s13578-019-0310-2) contains supplementary material, which is available to authorized users.

## Background

Thyroid cancer is the most common malignant endocrine tumor. The incidence of thyroid cancer is rapidly increasing worldwide and annually increasing in China, mainly due to improvements in thyroid cancer diagnostic technologies, especially increased detection of thyroid microcarcinoma [1]. According to data from the National Cancer Institute, thyroid cancer is responsible for 567,000 cancer cases worldwide, ranking ninth in tumor incidence. The global incidence rate of 10.2 per 100,000 in women is 3 times higher than that in men. Thyroid cancer accounts for 5.1% of the total estimated cancer burden in females in 2018 [2]. Most thyroid cancer cases originate in the follicular epithelium and can be divided into papillary thyroid cancer (PTC), follicular thyroid cancer (FTC) and anaplastic thyroid cancer (ATC) according to the pathologic types. Among all thyroid cancer cases, over 80% are PTC, which is common in young women and children, and FTC and ATC accounts for only 15% and less than 5% of cases, respectively [3]. Differentiated thyroid carcinoma (DTC), including PTC and FTC, has a favorable prognosis due to slow growth, well differentiation and low-grade malignancy. However, approximately 20–30% of PTC patients will develop recurrence, and patients with recurrent disease have poor prognoses. Notably, in contrast, ATC is characterized by poor differentiation and is notorious for its aggressive clinical behavior and tendency to rapidly metastasize and develop intrinsic resistance to chemotherapy, making it one of the most malignant tumors [4]. Even with aggressive therapy, the 5-year survival rate of ATC patients is less than 10%. Due to the lack of effective biomarkers for well-differentiated and poorly differentiated thyroid carcinoma, inappropriate excessive treatments have been used in clinical therapy. Therefore, there is an urgent need to investigate the molecular mechanisms underlying the development and progression of thyroid cancer for identifying potential molecular biomarkers to distinguish various subtypes of thyroid cancer and develop effective therapeutic strategies.

The insulin-like growth factor (IGF) signaling pathway is important for regulating cell proliferation, differentiation and apoptosis in mammals and for promoting tumor development and progression. Insulin-like growth factor binding proteins (IGFBPs) affect the interaction between IGF and its receptor and regulate the activity of the IGF signaling pathway. The IGFBP family consists of seven structurally homologous proteins (IGFBP1–IGFBP7), which bind insulin-like growth factor I (IGF-I) and insulin-like growth factor 2 (IGF-II) with high affinity, carry and transfer these IGFs, and extend their half-lives, thus regulating the biological function of IGFs. Among the IGFBPs, IGFBP7, a soluble protein identified in normal meningeal cells and mammary epithelium cells in humans, has been found to bind IGFs with low affinity and to bind insulin with high affinity. In addition, IGFBP7 can participate in a variety of pathophysiological processes, such as cell growth, differentiation, development, senescence and carcinogenesis, independent of the IGF signaling pathway. IGFBP7 is highly expressed in senescent breast epithelial cells [5]. Accumulating evidence suggests that IGFBP7 serves as a tumor suppressor in various types of cancers. Downregulation and even deletion of IGFBP7 have been found in breast cancer, lung cancer, bladder cancer, colorectal cancer, prostate cancer and melanoma, and low levels of IGFBP7 in tumor tissues are correlated with poor prognosis [6–10]. Overexpression of IGFBP7 in lung cancer cells inhibits anchorage-independent growth and xenograft tumor growth [11]. Similar findings have been obtained in other cancer types, such as colon cancer, prostate cancer and melanoma [12–14]. However, the expression pattern of IGFBP7 and its biological function in various types of thyroid carcinoma have not been clearly elucidated.

In this study, we found that IGFBP7 expression is greatly decreased and even absent in follicular and anaplastic thyroid carcinoma compared to that in normal thyroid tissue, adenoma and classical PTC. Overexpression of IGFBP7 in two undifferentiated human anaplastic thyroid carcinoma cell lines, ARO and FRO, and one differentiated follicular human thyroid carcinoma cell line, WRO, significantly inhibits tumor proliferation both in vitro and in vivo, whereas knockdown of endogenous IGFBP7 in WRO cells substantially increases proliferation and tumor growth. Furthermore, IGFBP7 dramatically suppresses the phosphorylation-mediated activation and kinase activity of AKT, causing upregulation of cyclin-dependent kinase (CDK) inhibitors, p27^Kip1^ and p21^Cip1^, and induction of G1/S cell cycle arrest. Taken together, our findings suggest that IGFBP7 plays an important tumor-suppressive role in human thyroid cancer, especially in FTC and ATC subtypes and may represent a promising biomarker and therapeutic target for the disease.

## Results

### IGFBP7 expression is downregulated in the FTC and ATC subtypes of thyroid cancer

**Fig. 1:**
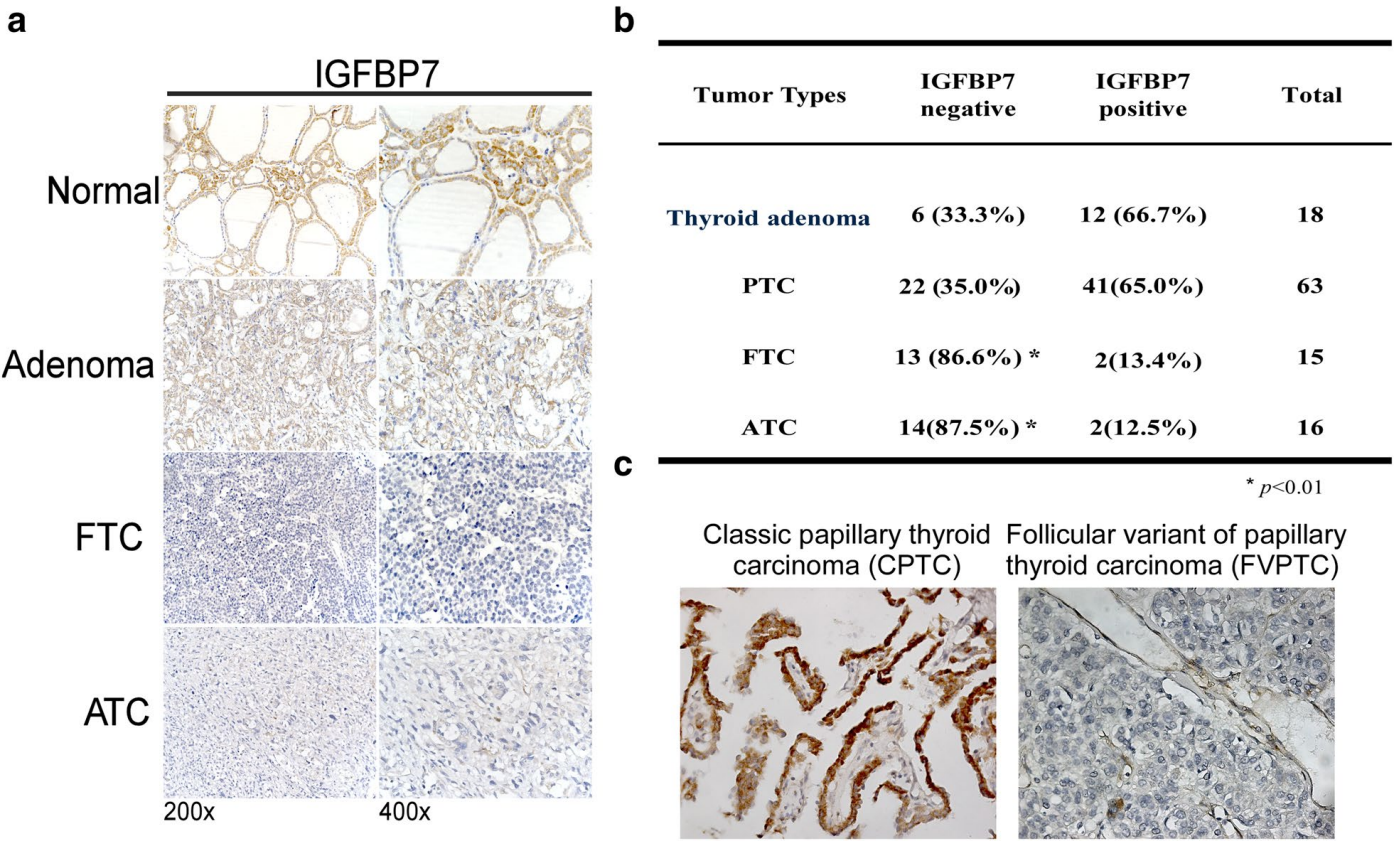
IGFBP7 is downregulated in human thyroid cancer samples. **a** Representative images of IGFBP7 immunostaining in normal thyroid tissue, thyroid adenoma and thyroid carcinoma specimens with different pathological characteristics. **b** Statistical analysis of the proportions of negative or positive IGFBP7 expression in clinical specimens of thyroid adenoma and thyroid carcinoma with different pathological subtypes. **c** Representative images of IGFBP7 immunostaining in two different papillary thyroid cancer (CPTC/FPTC) tissues

To determine the protein levels of IGFBP7 in different subtypes of thyroid tumors, we employed immunohistochemical staining in our collection of 112 paraffin-embedded, archived thyroid neoplasm specimens, including 18 cases of thyroid adenoma, 63 cases of papillary thyroid carcinoma (PTC), 15 cases of follicular thyroid carcinoma (FTC), and 16 cases of anaplastic thyroid carcinoma (ATC). As shown in Fig. 1a, b, positive expression of IGFBP7 was detected in 66.7% of thyroid adenoma tissues and 65.0% of PTC tissues, but IGFBP7 was detectable only in 13.4% of FTC and 12.5% of ATC tissues. Notably, classical papillary thyroid carcinoma (CPTC) tissues showed much strong IGFBP7 immunostaining, whereas follicular papillary thyroid carcinoma (FVPTC) tissues hardly showed IGFBP7 expression (Fig. 1c). These data showed that the expression of IGFBP7 was significantly lower or even absent in FTC and ATC as compared with that in normal thyroid tissues, benign thyroid tumors and classical PTC, suggesting that IGFBP7 may represent a valuable biomarker for well-differentiated and poorly differentiated thyroid carcinoma.

### IGFBP7 inhibits the proliferation of thyroid cancer cells in vitro

**Fig. 2:**
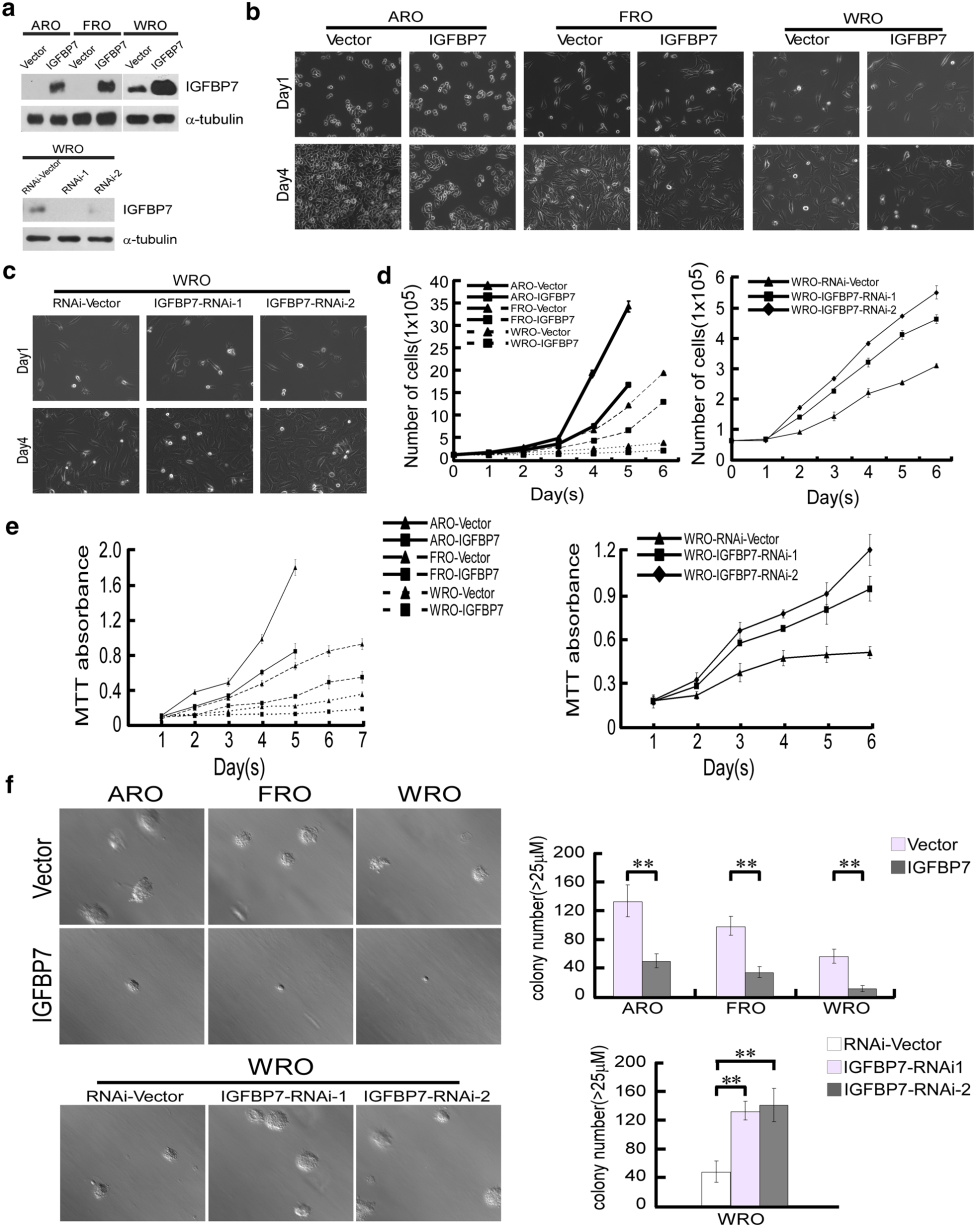
IGFBP7 exerts potent inhibitory effects on proliferation of ATC and FTC cell lines in vitro. **a** Western blotting assay validated the protein levels of IGFBP7 in ARO, FRO, and WRO cell lines overexpressed or silenced with IGFBP7. α-tubulin was used as a loading control. **b**–**d** Representative images and quantitation of IGFBP7-overexpressed or silenced cells at the indicated time points following initially plating the same number of cells. **e** MTT assay was conducted to measure the effect of IGFBP7 on the proliferation of the indicated cells at the indicated time points. **f** Representative micrographs (left) and relative quantification (right) of the indicated cells as evaluated by soft agar assay. All experiments were repeated three times with similar results. Data represent the mean ± S.D. of three independent experiments. A two-tailed Student’s t-test was used for statistical analysis (*P < 0.05, **P < 0.01)

To examine whether IGFBP7 is functionally involved in the development and progression of thyroid cancer, we used two undifferentiated human anaplastic thyroid carcinoma cell lines, ARO and FRO, both of which have undetectable levels of IGFBP7, to ectopically overexpress IGFBP7, and we employed one differentiated follicular human thyroid carcinoma cell line, WRO, which shows moderate IGFBP7 expression, to ectopically overexpress and knockdown IGFBP7 (Fig. 2a). After plating the same number of cells, we observed that vector control cells grew to reach 80–90% confluence within 3 or 5 days, whereas IGFBP7-overexpressing cells reached approximately 20% confluence. Moreover, knockdown of IGFBP7 with two specific shRNAs in WRO cells exhibited a more than twofold increase in cell number compared with that in the vector control cells (Fig. 2b, c). Consistent with these results, both cell counting and MTT assays revealed that while ectopic overexpression of IGFBP7 significantly suppressed proliferation of thyroid cancer cells, silencing of IGFBP7 accelerated thyroid cancer cell growth (Fig. 2d, e). Furthermore, we assessed the effect of IGFBP7 deregulation on anchorage independent growth using soft agar assay, and the results showed that thyroid cancer cells overexpressing IGFBP7 formed fewer and smaller cell colonies in the 3-D setting, and IGFBP7-silenced thyroid cancer cells produced more and larger colonies (Fig. 2f). Similarly, colony formation assays showed that the ability of cells to form cellular colonies in the 2-D setting was significantly suppressed upon IGFBP7 overexpression in comparison with that of their corresponding vector-control cells, whereas silencing of IGFBP7 apparently promoted formation of cellular colonies (Additional file 1: Figure S1). These results suggest that IGFBP7 strongly inhibits the proliferation of FTC and ATC cells in vitro.

### IGFBP7 suppresses tumor growth of thyroid cancer cells in vivo

**Fig. 3:**
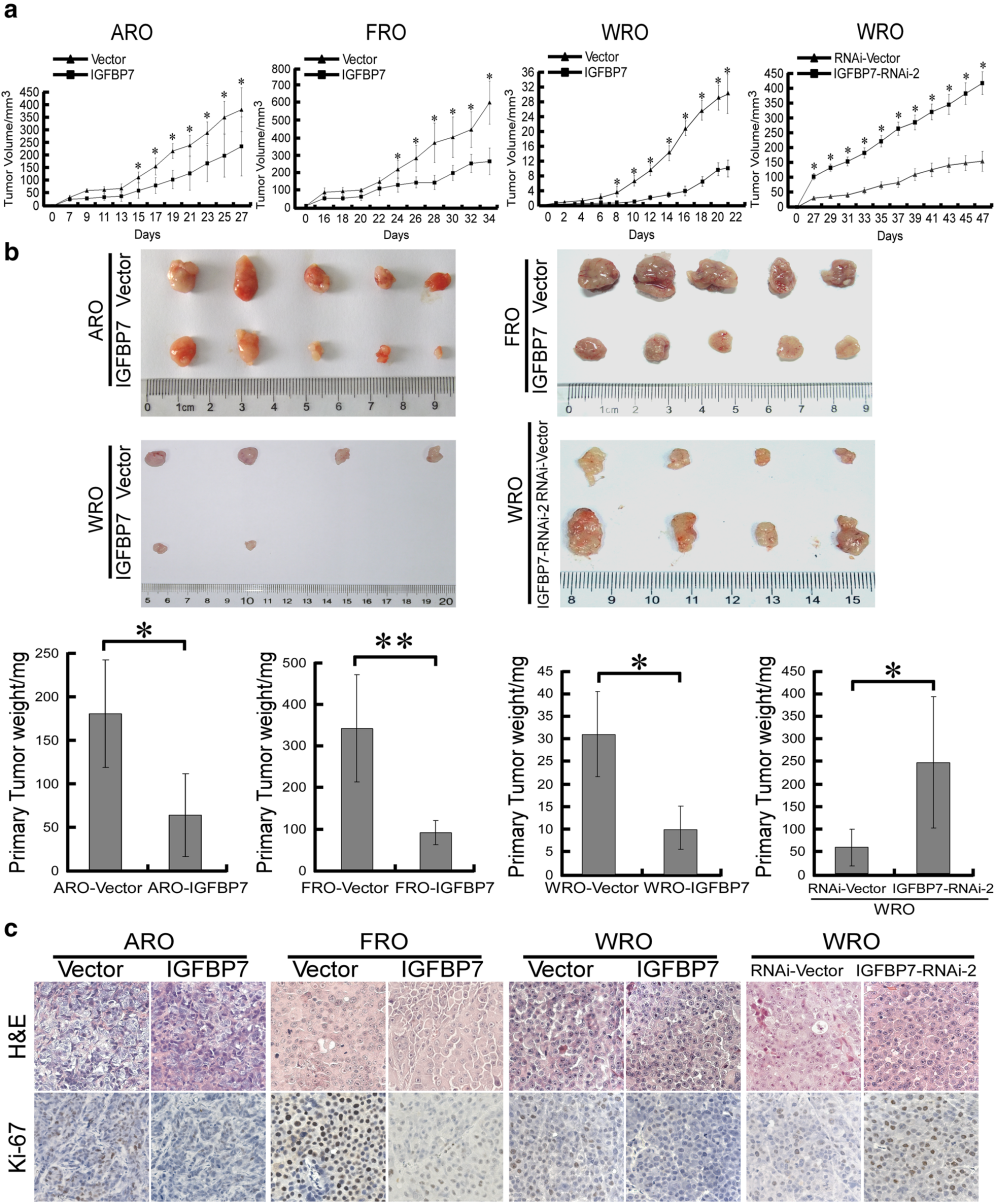
IGFBP7 suppresses tumor growth in vivo. **a** Quantitative analysis of tumor volumes of the indicated cell xenografts at the indicated time points. **b** Representative image of subcutaneous tumors isolated from nude mice at the experimental endpoint (upper) and quantitative analysis of tumor weights of the indicated cell xenografts (lower). **c** Tumor tissue sections from the indicated cell xenografts were prepared and proceeded for H&E staining and anti-Ki-67 immunostaining. A two-tailed Student’s t-test was used for statistical analysis (*P < 0.05, **P < 0.01)

Next, we assessed whether IGFBP7 inhibits growth of thyroid cancer cell-derived tumors using subcutaneous xenografts. As shown in Fig. 3a, IGFBP7-overexpressing ARO, FRO and WRO cells grew much more slowly than their corresponding vector control cells following subcutaneous inoculation, while IGFBP7-silenced WRO cells showed a much higher tumor growth rate than vector control WRO cells. In parallel, at the experimental endpoint, excised tumors formed by IGFBP7-overexpressing ARO, FRO and WRO cells had smaller size and lower weight than tumors formed by control cells, and IGFBP7 silencing produced the opposite effect (Fig. 3b). Notably, while four out of five mice inoculated with vector control WRO cells presented detectable tumor xenografts, only two out of five mice inoculated with WRO-IGFBP7 cells presented tumor growth (Fig. 3b), suggesting that IGFBP7 overexpression apparently suppressed not only the tumor growth rate, but also the tumorigenicity of thyroid cancer cells. Furthermore, the histological types of respective subcutaneous tumors were confirmed by H&E staining (Fig. 3c). Moreover, the proportion of proliferative Ki67-positive cells was significantly decreased in tumor tissues of IGFBP7-overexpressing xenografts and notably increased in IGFBP7-silenced tumor tissues (Fig. 3c). Taken together, these data suggest that IGFBP7 strongly suppresses the growth of ATC and FTC cell-derived tumors in vivo and that IGFBP7 represents a potent tumor suppressor preventing the development and progression of thyroid cancer.

### IGFBP7 induces the expression of the cell- cycle inhibitors p21^Cip1^ and p27^Kip1^, leading to cell cycle arrest

**Fig. 4:**
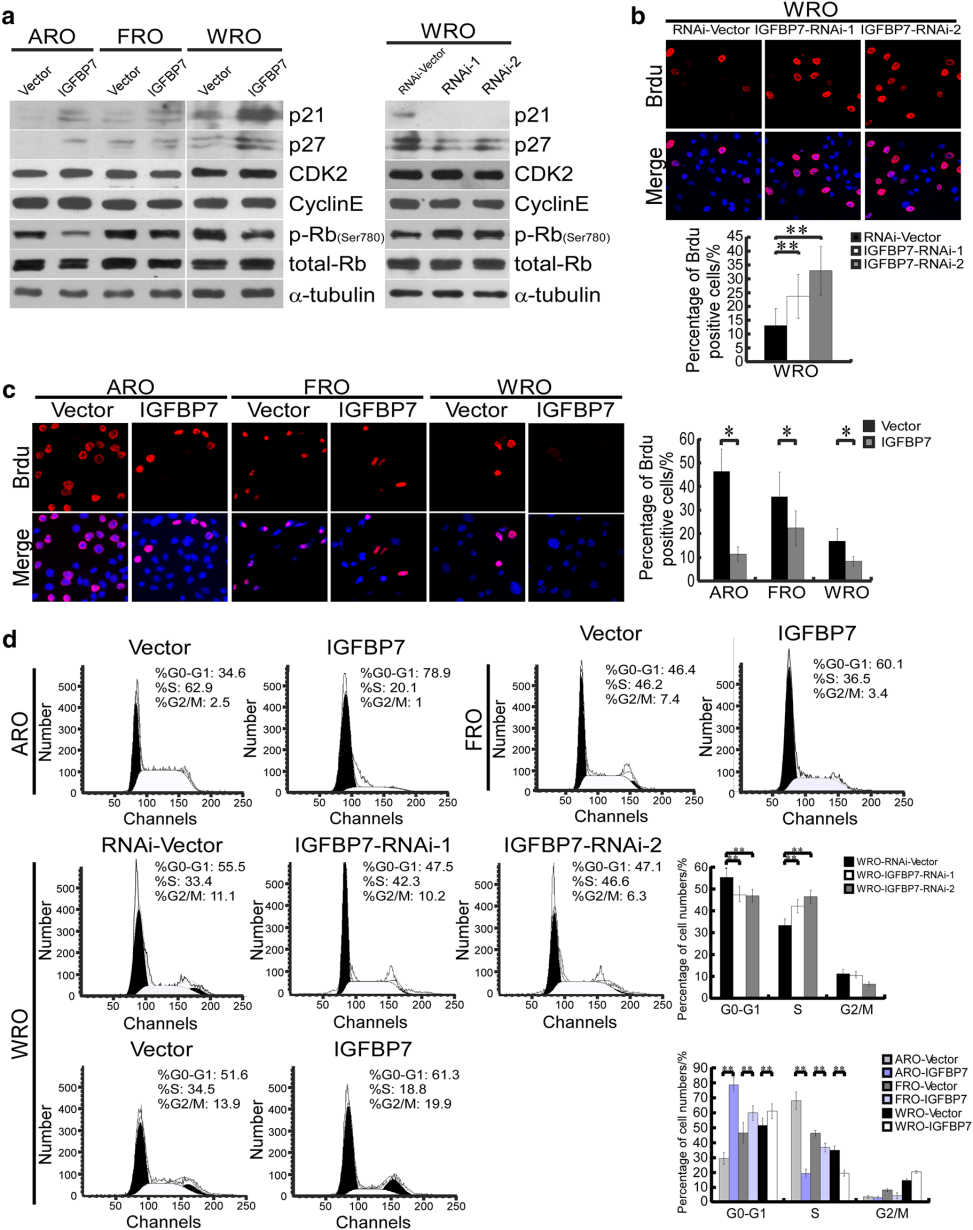
IGFBP7 upregulates expression of p21^Cip1^ and p27^Kip1^ and induces G1-S arrest. **a** Western blotting analysis of levels of p21^Cip1^, p27^Kip1^, CDK2, cyclin E, phosphorylated Rb (p-Rb, Ser780) and total Rb in the indicated cells. α-tubulin was used as a loading control. **b**, **c** Representative micrographs (upper) and quantification of the proportions (lower) of BrdU incorporating cells in IGFBP7-overexpressed or-silenced cells, as well as corresponding vector-control cells. **d** Flow cytometric analysis of the indicated thyroid cancer cell lines with IGFBP7 overexpression or knock down. Data represent the mean ± S.D. of three independent experiments. * *P* < 0.05, ** *P* < 0.01

To reveal the mechanism underlying the anti-proliferative effect of IGFBP7 on thyroid cancer, we analyzed the expression levels of cell cycle regulators. As shown in Fig. 4a, ectopic expression of IGFBP7 dramatically upregulated the levels of two cell- cycle inhibitors, namely, p21^Cip1^ and p27^Kip1^, accompanied by decreased levels of phosphorylated Rb, although the expression of CDK2 and Cyclin E was not altered. Additionally, the opposite results were observed in IGFBP7-silenced cells, indicating that IGFBP7 might inhibit cell- cycle progression in thyroid cancer cells. As it is well known that p21^Cip1^ and p27^Kip1^ play important inhibitory roles in the transition from G1 to S phase, we further assessed whether IGFBP7 influences G1/S progression using the 5-bromo-2-deoxyuridine (BrdU) incorporation assays and flow cytometry analyses. ARO and FRO cell lines were synchronized by serum starvation for 36 h and then induced to re-enter the cell cycle by the addition of 10% serum. As shown in Fig. 4b, the proportion of BrdU-positive ARO-IGFBP7 (10.6%), FRO-IGFBP7 (21.2%) and WRO-IGFBP7 (7.7%) cells were much lower than that of their corresponding vector control cells (44.4%, 34.0% and 16.0% for ARO, FRO and WRO cells, respectively), whereas BrdU-positive signals were prominently enhanced in IGFBP7-silenced cells (26.7% and 37.1% for WRO cells transduced with the two IGFBP7-targeting shRNAs) in contrast to vector control WRO cells (15.7%) (Fig. 4b). Consistent with these results, flow cytometry analysis demonstrated that ectopic expression of IGFBP7 in ATC and FTC cell lines increased the percentage of cells in the G0/G1 phase of the cell cycle and decreased the percentage of cells in the S phase (Fig. 4c). In contrast, IGFBP7 silencing facilitated cell cycle progression through G1 to S phase (Fig. 4c). Taken together, these data suggest that IGFBP7 is able to induce the expression of the critical cell-cycle inhibitors p21^Cip1^ and p27^Kip1^, causing G1-S phase arrest during the cell cycle and subsequent growth inhibition.

### IGFBP7 suppresses the phosphorylation-mediated activation and kinase activity of AKT in thyroid cancer

**Fig. 5:**
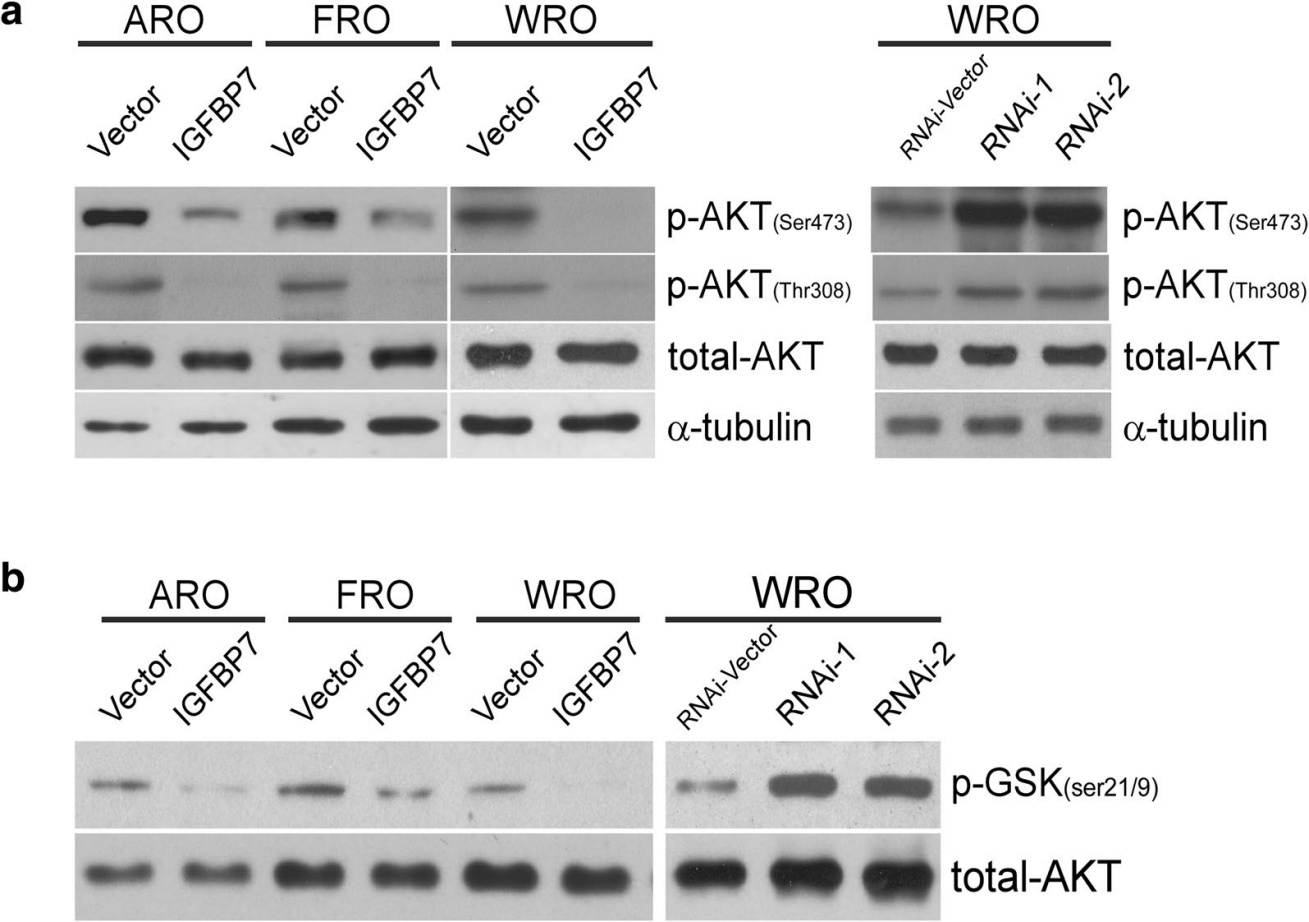
IGFBP7 inhibits the phosphorylation and kinase activity of AKT. **a** Western blotting analysis of phosphorylated AKT (Ser 473 and Thr 308) and total AKT in the indicated cells. α-tubulin was used as a loading control. **b** The AKT kinase assay measuring AKT activity in vitro was performed. Western blotting analysis of p-GSK-3β (Ser21/9) and total AKT in the indicated cells. All data were obtained from three independent experiments with similar results

Numerous studies have illustrated that AKT crucially regulates the expression of p21^Cip1^ and p27^Kip1^ and cell cycle progression. We thus further investigated the effect of IGFBP7 on both phosphorylation levels and kinase activity of AKT. As expected, overexpression of IGFBP7 markedly reduced the phosphorylation of AKT (Ser473 and Thr308) in various thyroid cancer cells, whereas silencing of IGFBP7 enhanced AKT phosphorylation (Ser473 and Thr308) (Fig. 5a), indicating that IGFBP7 suppresses phosphorylation-mediated activation of AKT. Meanwhile, an in vitro AKT kinase activity assay was employed to verify the inhibitory effect of IGFBP7 on AKT activity. As shown in Fig. 5b, the kinase activity assay demonstrated that silencing of IGFBP7 in various thyroid cancer cells markedly promoted, whereas overexpression of IGFBP7 suppressed, endogenous AKT activity, as demonstrated by the phosphorylation levels of a common AKT substrate, GSK3β. Collectively, our data suggest that IGFBP7 suppresses proliferation through the inhibition of both phosphorylation activation and kinase activity of AKT in thyroid cancer.

## Discussion

Thyroid cancer is the most commonly diagnosed endocrine malignancy. Thyroid cancer is an excellent example of how technological advancements in diagnostic imaging have led to the detection of indolent disease. Although an increase in incidence is largely confined to the more indolent histological subtype and to early tumor stages, thyroid cancer is a disease with several diverse subtypes. The various subtypes, mainly PTC, FTC and ATC, are different in their unique biology, natural history, prognosis, and available therapeutic options [15–20]. Although PTC patients have a favorable prognosis, approximately 20–30% of PTC patients will develop recurrence and recurrent diseases have poor therapeutic responsiveness similar to undifferentiated thyroid carcinomas [21–23]. Molecular studies are being focused on better characterizing the various subtypes of thyroid cancer and improving diagnosis by fine-needle aspiration biopsy. However, definite markers for the different subtypes of thyroid cancer are yet to be identified. Thyroid cancer pathology has generally been limited to the identification of features that predict aggressiveness, within the accepted “malignant” categories of PTC, ATC and FTC. Notably, our current study demonstrated that the expression of IGFBP7 in FTC and ATC is significantly lower or even absent compared with that in normal thyroid tissues, benign thyroid tumors and PTC. Furthermore, CPTC shows strong immunostaining for IGFBP7, whereas FVPTC hardly shows detectable levels of IGFBP7. These data suggest that IGFBP7 may be a valuable biomarker for FTC and ATC, both of which have malignant features. Similarly, IGFBP7 is almost undetectable in ATC cell lines and moderately expressed in FTC cell lines. Moreover, overexpression of IGFBP7 potently suppresses, and silencing of IGFBP7 accelerates proliferation and tumor growth of both ATC and FTC cell lines, suggesting the important role for the loss of IGFBP7 expression during thyroid cancer development and progression. Notably, many newly diagnosed cases of thyroid cancer present only as microcancerous lesions, making it difficult to discern the histological types and to choose appropriate treatment [24]. Since IGFBP7 is a secretory protein that could be easily detected in clinical specimens such as blood, it is of great interest to detect levels of serum IGFBP7 in patients with benign thyroid adenoma or malignant thyroid carcinoma of various subtypes, such that serum IGFBP7 levels might be used as valuable biomarkers for the early diagnosis and clinical therapy of thyroid neoplasms.

Different types of thyroid cancer have unique gene mutations, suggesting that different molecular mechanisms may be related to the development and progression of thyroid cancer. Point mutations in the BRAF or RAS gene or RET/PTC rearrangements, all of which lead to constitutive activation of the mitogen-activated protein kinase (MAPK) signaling pathway, can be found in approximately 70% of thyroid cancer patients. Nevertheless, it is not common to have two or more mutations in the same patient at the same time [25, 26]. Among these mutations, BRAFV600E is considered to be the marker for the diagnosis and prognosis of papillary thyroid cancer and is closely related to extrathyroidal infiltration, lymph node metastasis, clinical stage, recurrence and mortality of thyroid cancer [27]. It has been reported that IGFBP7 inhibits the BRAF-MEK-ERK pathway in an autocrine or paracrine manner and causes apoptosis in BRAF mutation-positive melanoma cells. However, we detected the phosphorylated levels of MEK and ERK in thyroid cancer cells with overexpression or knockdown of IGFBP7 and found that IGFBP7 did not impact on the BRAF-MEK-ERK pathway (data not shown). In parallel, the various thyroid cancer tissues collected in our study showed loss of IGFBP7 expression regardless of BRAF status. In fact, an increasing number of studies suggest that hyperactivation of the PI3K/AKT signaling pathway is as important as the BRAF-MEK-ERK pathway during tumor development and progression. For example, Maria et al. demonstrated that the PI3K/AKT signaling pathway is activated in more than 50% of thyroid cancer cases [28]. Several reports have shown that gene mutations in the PI3K/AKT pathway are mainly present in poorly differentiated thyroid cancer tissues [29–31], suggesting that the activation of the PI3K/AKT signaling pathway may play an important role in the development and progression of thyroid cancer. In support of this notion, our current study reveals that IGFBP7 potently suppresses the growth of ATC and FTC cell-derived tumors and inhibits both phosphorylation-mediated activation and kinase activity of AKT. Therefore, our data suggest that the loss of IGFBP7 causes overactivation of the AKT signaling pathway, an effect essentially equivalent to that of the well-documented genetic mutations causing overactivation of the BRAF-MEK-ERK pathway. Notably, as IGFBP7 is a secreted protein and has low affinity for IGF, how IGFBP7 blocks the activation of AKT signaling remains to be investigated.

From a biological point of view, tumors, characterized by hyperproliferation, disrupted cell cycle and dysregulated of signal transduction pathways, are indicative of progressive disorders in cell cycle regulation. In normal cells, each stage of the cell cycle is controlled by a series of regulatory mechanisms to ensure that the cell cycle is carried out in a strict and orderly fashion. Three key classes of regulatory molecules, namely, cyclins, cyclin-dependent kinases (CDKs) and cyclin-dependent kinase inhibitors (CKIs), determine the progress of a cell through the cell cycle. Therefore, it is important to determine which types of these regulatory molecules are involved under a certain condition. In this study, we found that ectopic expression of IGFBP7 dramatically upregulates the levels of p27^Kip1^ and p21^Cip1^ and that the silencing of IGFBP7 causes the opposite effects in ATC and FTC cells. Notably, the cell cycle regulator p27^Kip1^ is a tumor-suppressive protein that broadly inhibits CDKs and plays an important role in resting cells in the G1 phase during the cell cycle [32]. P21^Cip1^ which halts the cell cycle in the G1 phase by binding to and inactivating cyclin-CDK complexes is also a negative regulator of cell cycle progression and a typical tumor suppressor gene [33]. After the inhibition of protein kinase activity in cyclin-CDK complexes by p27^Kip1^ and p21^Cip1^, Rb is in a state of hypophosphorylation. Hypophosphorylated Rb can bind the critical transcription factor E2F, making it lose its function and causing cell cycle arrest at G1 phase [34]. Indeed, our data demonstrate that IGFBP7 overexpression significantly increases the percentage of cells in the G0/G1 phase and reduces the percentage of cells in the S phase of the cell cycle, and IGFBP7 silencing causes the opposite effects in ATC and FTC cells. Thus, we propose that IGFBP7 induces G1-S phase arrest during the cell cycle by upregulating the expression of two critical CKIs, namely, p27^Kip1^ and p21^Cip1^, to potently inhibit the proliferation of ATC and FTC cells. Notably, the AKT signaling pathway has been widely reported to decrease the expression of p27^Kip1^ and p21^Cip1^ through inactivating its downstream substrates, such as FOXO transcriptional factors, and to promote cell cycle progression [35–39]. Therefore, whether and how IGFBP7 inhibits the AKT signaling pathway to upregulate the expression of p27^Kip1^ and p21^Cip1^ in thyroid cancer requires further investigation.

Although IGFBP7 is present in almost all normal tissues, IGFBP7 is downregulated or even absent in a variety of tumor types, including breast cancer, lung cancer, bladder cancer, colorectal cancer, prostate cancer, melanoma and thyroid cancers [40–42]. Similar to that for many other tumor suppressors, the downregulated expression of IGFBP7 in tumor tissues is mainly ascribed to genetic and epigenetic regulation. It was reported that loss of heterozygosity (LOH) causes loss of expression of IGFBP7 in breast cancer [6]. Accumulating evidence has demonstrated that hypermethylation of the IGFBP7 gene promoter leads to its loss of expression. Moreover, tumor cells lacking IGFBP7 expression could be restored by treatment with the DNA methylation inhibitor 5-aza-2′-deoxycytidine [7, 43]. Therefore, it is of great interest to further investigate whether the significant downregulation of IGFBP7 in ATC and FTC tissues, but not in classical PTC tissues, is caused by LOH or hypermethylation.

## Conclusions

Taken together, results from the present study showed that the expression of IGFBP7 is significantly downregulated and even absent in thyroid follicular carcinoma and anaplastic carcinoma tissues. IGFBP7 might act as a tumor suppressor by inhibiting the proliferation of thyroid cancer cells via the downregulation of the activity of AKT, leading to an increase in the expression of the cell cycle inhibitors p27^Kip1^ and p21^Cip1^.

## Methods

### Tissue specimens

This study was conducted with a cohort of paraffin-embedded sections of 112 thyroid tumor patients, who had undergone surgical resection in the first affiliated hospital of Sun Yat-sen university during the period of 2005 to 2010. The histologic types of the above tumor samples were as the following: 18 cases of thyroid adenomas, 63 cases of papillary thyroid cancer, 15 cases of follicular thyroid cancer, 16 cases of anaplastic thyroid cancer.

### Cell cultures

The undifferentiated thyroid cancer cell lines FRO and ARO, follicular thyroid cancer cell lines WRO were gifted from the surgical laboratory of the Chinese University of Hong Kong, and maintained in DMEM medium (Invitrogen, Carlsbad, CA) supplemented with 10% fetal bovine serum (HyClone, Logan, UT) and 1% penicillin/streptomycin (Invitrogen, Carlsbad, CA), according to the protocols of previous reports [44]. All cell lines were authenticated by short tandem repeat fingerprinting at Medicine Laboratory of Forensic Medicine Department of Sun Yat-Sen University (Guangzhou, China), and were free of mycoplasma contamination.

### Plasmids and transfection

IGFBP7 expression plasmid was generated by PCR sub-cloning of human IGFBP7 coding sequence into retroviral transfer plasmid pMSCV to generate plasmid pMSCV-retro-puro-IGFBP7. For depletion of IGFBP7, two human shRNA sequences were cloned into the pSuper-retro-puro plasmid to generate pSuper-retro-IGFBP7-RNAi(s), respectively. Retroviral production and infection were performed as previously described [45], and stable cell lines were selected by treatment with 0.5 μg/ml puromycin for 10–14 days, beginning at 48 h after infection.

### Western blotting analysis

Western blotting analysis was performed according to a standard method previously described [45], using anti-IGFBP7, anti-p21, anti-p27, anti-CDK2, anti-Cyclin E, anti-phosphor-Rb (Ser780) and anti-Rb, anti-AKT, anti-phosphor-AKT (Ser473) and anti-phosphor-AKT (Thr308) antibodies. Blotted membranes were stripped and re-blotted with an anti-α-tubulin mouse monoclonal antibody (Sigma–Aldrich, St. Louis, MO) used as a loading control.

### 3-(4,5-Dimethyl-2-thiazolyl)-2,5-diphenyl-2H-tetrazolium bromide (MTT) assay

According to previous studies [46], cell viability was determined using an MTT assay. The cells were seeded at a density of 5 × 10^3^ cells per well in 96-well plates. Subsequently at 1, 2, 3, 4, 5 and 6 days, 20 μl MTT (Sigma–Aldrich, St. Louis, MO) was added to each well and incubated for 4 h. The culture medium was removed, and 200 μl dimethyl sulfoxide (DMSO) (Amresco, Solon, Ohio) was added to each well. The plates were then shaken at room temperature for 10 min, and the absorbance of stained cells was measured at 570 nm, with 655 nm as the reference wavelength. Each experiment was performed in triplicates and repeated for three times.

### Colony formation and soft agar assays

For the 2-D colony formation assay, cells were plated into 6-well plates at the density of 200–500 per well. The cells were allowed to grow for 7–12 days and stained with crystal violet. The plates were photographed and the numbers of colonies formed by indicated cells were quantified using the Quantity One software package (Bio-Rad, Hercules, CA). For the soft agar assays, one or three thousand cells were trypsinized and suspended in 2 ml of culture medium containing 0.3% agar (Sigma). The agar–cell mixture was grown on top of 1% agar contained in culture medium. After 10 days, viable cellular colonies larger than the indicated diameter were counted. Each experiment was repeated for three times.

### Bromodeoxyuridine labeling and immunofluorescence

Cells were plated on coverslips (Fisher, Pittsburgh, PA). After 24 h, cells were incubated with bromodeoxyuridine (BrdU) for 1 h and stained with anti-BrdU antibody (Upstate, Temecula, CA) according to the manufacturer’s instruction. Immunostaining images were acquired under a laser scanning microscope (Axioskop 2 plus, Carl Zeiss Co. Ltd., Jena, Germany). Each experiment was repeated for three times.

### Flow cytometry

Cells were harvested, washed with cold PBS and processed for cell cycle analysis using flow cytometry. Briefly, the cells were fixed in 75% ethanol and stored at − 20 °C for later analysis. The fixed cells were centrifuged at 1000 rpm and washed with cold PBS twice. RNase A (20 μg/ml final concentration) and propidium iodide staining solution (50 μg/ml final concentration) was added to the cells and incubated for 30 min at 37 °C in the dark. Fifty thousand cells were analyzed using a FACSC alibur instrument (BD Biosciences, San Jose, CA) equipped with CellQuest 3.3 software. ModFit LT 3.1 trial cell cycle analysis software was used to determine the percentage of cells in the different phases of the cell cycle. Each experiment was repeated for three times.

### Immunohistochemical analysis (IHC)

A total of 112 paraffin-embedded thyroid tumor samples described above were used in the current study. For the use of these clinical materials for research purposes, prior patients’ consents and approval from the Institutional Research Ethics Committee were obtained. After deparaffinization, sections were immunostained using anti-IGFBP7 monoclonal antibody. The resultant immunostaining images were captured using the AxioVision Rel.4.6 computerized image analysis system (Carl Zeiss, Oberkochen, Germany). The procedure was carried out according to previously described methods [47]. The degree of immunostaining of formalin-fixed, paraffin-embedded sections was reviewed and scored independently by two investigators, based on both the proportion of positively-stained tumor cells and the intensity of staining. The observers were blinded to the histopathological features and patient data of the samples.

### AKT kinase activity detection

The AKT Kinase Assay kit was purchased from the Cell Signaling (Danvers, MA, # 9840). As instructed by the provided protocol, immobilized AKT antibody was used to immune-precipitate AKT from the indicated cell extracts [48]. Immune-precipitated pellets were then incubated in the Kinase Buffer containing GSK-3 fusion protein and cold ATP. Levels of GSK-3 phosphorylation using the anti-phospho-GSK-3α/β (Ser21/9) antibody were measured by western blotting and chemiluminescent detection. All the experimental steps were carried out strictly according to the instructions of the kit. Each experiment was repeated for three times.

### In vivo tumor growth assay

For the in vivo tumor growth assay, ARO-Vector and ARO-IGFBP7cells (2x10^6^ cells each), FRO-Vector and FRO-IGFBP7 cells (5 × 10^6^ cells each), WRO-Vector and WRO-IGFBP7 (8 × 10^7^ cells each), WRO-RNAi-Vector and WRO-IGFBP7-RNAi-2 (7 × 10^7^ cells each) were subcutaneously inoculated into the dorsal thighs of individual female BALB/C nude mice (6–7 weeks of age, 20–24 g). Tumor lengths (L) and widths (W) were measured every 2 days using a digital caliper, and tumor volumes were calculated using the equation *volume* (mm^3^) = L*W^2^/2. After 3–4 weeks, mice were anesthetized and sacrificed, and tumors were removed completely, photographed and weighed. The tumor growth curves were plotted and statistically analyzed. The measurement data were expressed as mean value plus standard deviation (X ± S). The comparison between the two groups was conducted by t test. Moreover, the tumors were sectioned for HE staining and immunostaining of Ki-67. Animal experiments were approved by the Ethical Committee of Sun Yat-sen University.

### Statistical analysis

All statistical analyses were performed with the SPSS 20.0 software package (IBM, Chicago, IL, USA). Comparative analysis between groups was performed with two-tailed paired Student’s *t* test for statistical significance. The Chi square test was used to analyze the relationship between IGFBP7 expression and pathological characteristics. All bars represent the mean ± SD derived from three independent experiments. *P* values < 0.05 were considered significant.

## Additional file

**Additional file 1: Figure S1.** IGFBP7 inhibits proliferation of thyroid cancer cells in vitro. (a) Representative micrographs (upper) and relative quantification (lower) of the indicated cells as evaluated by the colony formation assay. Data represent the mean ± S.D. of three independent experiments. A two-tailed Student’s t-test was used for statistical analysis (*P < 0.05, **P < 0.01).

## Data Availability

Please contact author for data requests.

## References

[1] Cabanillas ME, McFadden DG, Durante C (2016). Thyroid cancer. Lancet.

[2] Bray F, Ferlay J, Soerjomataram I, Siegel RL, Torre LA, Jemal A (2018). Global cancer statistics 2018: GLOBOCAN estimates of incidence and mortality worldwide for 36 cancers in 185 countries. CA Cancer J Clin.

[3] Amodeo C, Caglia P, Gandolfo L, Veroux M, Donati M, Imme A (2003). Undifferentiated carcinoma of the thyroid. Tumori.

[4] Molinaro E, Romei C, Biagini A, Sabini E, Agate L, Mazzeo S, Materazzi G, Sellari-Franceschini S, Ribechini A, Torregrossa L (2017). Anaplastic thyroid carcinoma: from clinicopathology to genetics and advanced therapies. Nat Rev Endocrinol.

[5] Swisshelm K, Ryan K, Tsuchiya K, Sager R (1995). Enhanced expression of an insulin growth factor-like binding protein (mac25) in senescent human mammary epithelial cells and induced expression with retinoic acid. Proc Natl Acad Sci USA.

[6] Burger AM, Zhang X, Li H, Ostrowski JL, Beatty B, Venanzoni M, Papas T, Seth A (1998). Down-regulation of T1A12/mac25, a novel insulin-like growth factor binding protein related gene, is associated with disease progression in breast carcinomas. Oncogene.

[7] Hwa V, Tomasini-Sprenger C, Bermejo AL, Rosenfeld RG, Plymate SR (1998). Characterization of insulin-like growth factor-binding protein-related protein-1 in prostate cells. J Clin Endocrinol Metab.

[8] Landberg G, Ostlund H, Nielsen NH, Roos G, Emdin S, Burger AM, Seth A (2001). Downregulation of the potential suppressor gene IGFBP-rP1 in human breast cancer is associated with inactivation of the retinoblastoma protein, cyclin E overexpression and increased proliferation in estrogen receptor negative tumors. Oncogene.

[9] Tennant MK, Vessella RL, Sprenger CC, Sikes RA, Hwa V, Baker LD, Plymate SR (2003). Insulin-like growth factor binding protein-related protein 1 (IGFBP-rP1/mac 25) is reduced in human prostate cancer and is inversely related to tumor volume and proliferation index in Lucap 23.12 xenografts. Prostate..

[10] Ruan WJ, Lin J, Xu EP, Xu FY, Ma Y, Deng H, Huang Q, Lv BJ, Hu H, Cui J (2006). IGFBP7 plays a potential tumor suppressor role against colorectal carcinogenesis with its expression associated with DNA hypomethylation of exon 1. J Zhejiang Univ Sci B.

[11] Chen Y, Pacyna-Gengelbach M, Ye F, Knosel T, Lund P, Deutschmann N, Schluns K, Kotb WF, Sers C, Yasumoto H (2007). Insulin-like growth factor binding protein-related protein 1 (IGFBP-rP1) has potential tumour-suppressive activity in human lung cancer. J Pathol.

[12] Chen RY, Chen HX, Jian P, Xu L, Li J, Fan YM, Tu YT (2010). Intratumoral injection of pEGFC1-IGFBP7 inhibits malignant melanoma growth in C57BL/6J mice by inducing apoptosis and down-regulating VEGF expression. Oncol Rep.

[13] Drivdahl R, Haugk KH, Sprenger CC, Nelson PS, Tennant MK, Plymate SR (2004). Suppression of growth and tumorigenicity in the prostate tumor cell line M12 by overexpression of the transcription factor SOX9. Oncogene.

[14] Ruan W, Xu E, Xu F, Ma Y, Deng H, Huang Q, Lv B, Hu H, Lin J, Cui J (2007). IGFBP7 plays a potential tumor suppressor role in colorectal carcinogenesis. Cancer Biol Ther.

[15] Bulliard JL, Chiolero A (2015). Screening and overdiagnosis: public health implications. Public Health Rev.

[16] Chiolero A, Paccaud F, Aujesky D, Santschi V, Rodondi N (2015). How to prevent overdiagnosis. Swiss Med wkly.

[17] Davies L, Ouellette M, Hunter M, Welch HG (2010). The increasing incidence of small thyroid cancers: where are the cases coming from?. Laryngoscope.

[18] Ho AS, Davies L, Nixon IJ, Palmer FL, Wang LY, Patel SG, Ganly I, Wong RJ, Tuttle RM, Morris LG (2015). Increasing diagnosis of subclinical thyroid cancers leads to spurious improvements in survival rates. Cancer.

[19] Moynihan R, Doust J, Henry D (2012). Preventing overdiagnosis: how to stop harming the healthy. BMJ.

[20] Welch HG, Black WC (2010). Overdiagnosis in cancer. J Natl Cancer Inst.

[21] Lin HC, Liou MJ, Hsu HL, Hsieh JC, Chen YA, Tseng CP, Lin JD (2016). Combined analysis of circulating epithelial cells and serum thyroglobulin for distinguishing disease status of the patients with papillary thyroid carcinoma. Oncotarget.

[22] Ito Y, Higashiyama T, Takamura Y, Kobayashi K, Miya A, Miyauchi A (2010). Clinical outcomes of patients with papillary thyroid carcinoma after the detection of distant recurrence. World J Surg.

[23] Dinneen SF, Valimaki MJ, Bergstralh EJ, Goellner JR, Gorman CA, Hay ID (1995). Distant metastases in papillary thyroid carcinoma: 100 cases observed at one institution during 5 decades. J Clin Endocrinol Metab.

[24] Schneider DF, Chen H (2013). New developments in the diagnosis and treatment of thyroid cancer. CA Cancer J Clin..

[25] Frattini M, Ferrario C, Bressan P, Balestra D, De Cecco L, Mondellini P, Bongarzone I, Collini P, Gariboldi M, Pilotti S (2004). Alternative mutations of BRAF, RET and NTRK1 are associated with similar but distinct gene expression patterns in papillary thyroid cancer. Oncogene.

[26] Soares P, Trovisco V, Rocha AS, Lima J, Castro P, Preto A, Maximo V, Botelho T, Seruca R, Sobrinho-Simoes M (2003). BRAF mutations and RET/PTC rearrangements are alternative events in the etiopathogenesis of PTC. Oncogene.

[27] Xing M (2005). BRAF mutation in thyroid cancer. Endocr Relat Cancer.

[28] Motti ML, Califano D, Troncone G, De Marco C, Migliaccio I, Palmieri E, Pezzullo L, Palombini L, Fusco A, Viglietto G (2005). Complex regulation of the cyclin-dependent kinase inhibitor p27kip1 in thyroid cancer cells by the PI3K/AKT pathway: regulation of p27kip1 expression and localization. Am J Pathol.

[29] Garcia-Rostan G, Costa AM, Pereira-Castro I, Salvatore G, Hernandez R, Hermsem MJ, Herrero A, Fusco A, Cameselle-Teijeiro J, Santoro M (2005). Mutation of the PIK3CA gene in anaplastic thyroid cancer. Can Res.

[30] Hou P, Liu D, Shan Y, Hu S, Studeman K, Condouris S, Wang Y, Trink A, El-Naggar AK, Tallini G (2007). Genetic alterations and their relationship in the phosphatidylinositol 3-kinase/Akt pathway in thyroid cancer. Clin Cancer Res.

[31] Ricarte-Filho JC, Ryder M, Chitale DA, Rivera M, Heguy A, Ladanyi M, Janakiraman M, Solit D, Knauf JA, Tuttle RM (2009). Mutational profile of advanced primary and metastatic radioactive iodine-refractory thyroid cancers reveals distinct pathogenetic roles for BRAF, PIK3CA, and AKT1. Cancer Res.

[32] Polyak K, Lee MH, Erdjument-Bromage H, Koff A, Roberts JM, Tempst P, Massague J (1994). Cloning of p27Kip1, a cyclin-dependent kinase inhibitor and a potential mediator of extracellular antimitogenic signals. Cell.

[33] Pestell RG, Albanese C, Reutens AT, Segall JE, Lee RJ, Arnold A (1999). The cyclins and cyclin-dependent kinase inhibitors in hormonal regulation of proliferation and differentiation. Endocr Rev.

[34] Serres MP, Besson A (2009). Critical role for p27 in mediating the antitumoral activity of a proteasome inhibitor. Med Sci.

[35] Adachi M, Osawa Y, Uchinami H, Kitamura T, Accili D, Brenner DA (2007). The forkhead transcription factor FoxO1 regulates proliferation and transdifferentiation of hepatic stellate cells. Gastroenterology.

[36] Aoki M, Jiang H, Vogt PK (2004). Proteasomal degradation of the FoxO1 transcriptional regulator in cells transformed by the P3k and Akt oncoproteins. Proc Natl Acad Sci USA.

[37] Dijkers PF, Medema RH, Pals C, Banerji L, Thomas NS, Lam EW, Burgering BM, Raaijmakers JA, Lammers JW, Koenderman L (2000). Forkhead transcription factor FKHR-L1 modulates cytokine-dependent transcriptional regulation of p27(KIP1). Mol Cell Biol.

[38] Nakamura N, Ramaswamy S, Vazquez F, Signoretti S, Loda M, Sellers WR (2000). Forkhead transcription factors are critical effectors of cell death and cell cycle arrest downstream of PTEN. Mol Cell Biol.

[39] Seoane J, Le HV, Shen L, Anderson SA, Massague J (2004). Integration of Smad and forkhead pathways in the control of neuroepithelial and glioblastoma cell proliferation. Cell.

[40] Garcia-Rostan G, Camp RL, Herrero A, Carcangiu ML, Rimm DL, Tallini G (2001). Beta-catenin dysregulation in thyroid neoplasms: down-regulation, aberrant nuclear expression, and CTNNB1 exon 3 mutations are markers for aggressive tumor phenotypes and poor prognosis. Am J Pathol.

[41] Miyake N, Maeta H, Horie S, Kitamura Y, Nanba E, Kobayashi K, Terada T (2001). Absence of mutations in the beta-catenin and adenomatous polyposis coli genes in papillary and follicular thyroid carcinomas. Pathol Int.

[42] Murphy M, Pykett MJ, Harnish P, Zang KD, George DL (1993). Identification and characterization of genes differentially expressed in meningiomas. Cell Growth Differ.

[43] Burger AM, Zhang X, Seth A (1998). Detection of novel genes that are up-regulated (Di12) or down-regulated (T1A12) with disease progression in breast cancer. Eur J Cancer Prev.

[44] Yang Y, Liu L, Cai JC, Wu JH, Guan HY, Zhu X, Yuan J, Li MF (2014). DEPDC1B enhances migration and invasion of non-small cell lung cancer cells via activating Wnt/beta-catenin signaling. Biochem Biophys Res Commun.

[45] Cai J, Guan H, Fang L, Yang Y, Zhu X, Yuan J, Wu J, Li M (2013). MicroRNA-374a activates Wnt/beta-catenin signaling to promote breast cancer metastasis. J Clin Invest.

[46] Liu L, Wu S, Yang Y, Cai J, Zhu X, Wu J, Li M, Guan H (2016). SOSTDC1 is down-regulated in non-small cell lung cancer and contributes to cancer cell proliferation. Cell Bio sci.

[47] Jiang L, Lin C, Song L, Wu J, Chen B, Ying Z, Fang L, Yan X, He M, Li J (2012). MicroRNA-30e* promotes human glioma cell invasiveness in an orthotopic xenotransplantation model by disrupting the NF-kappaB/IkappaBalpha negative feedback loop. J Clin Invest.

[48] Maddika S, Chen JJ (2009). Protein kinase DYRK2 is a scaffold that facilitates assembly of an E3 ligase. Nat Cell Biol.

