# Correction to: IGFBP7 inhibits cell proliferation by suppressing AKT activity and cell cycle progression in thyroid carcinoma

**DOI:** 10.1186/s13578-019-0319-6

**Published:** 2019-07-15

**Authors:** Le Zhang, Rong Lian, Jingjing Zhao, Xianming Feng, Runyi Ye, Lingxiao Pan, Jueheng Wu, Mengfeng Li, Yongbo Huang, Junchao Cai

**Affiliations:** 1 Key Laboratory of Tropical Disease Control, Ministry of Education, Sun Yat-sen University, 74 Zhongshan Er Road, Guangzhou, 510080 Guangdong China; 2 Department of Microbiology, Zhongshan School of Medicine, Sun Yat-sen University, Guangzhou, 510080 Guangdong China; 3 Department of Cardiology, The First Affiliated Hospital, Sun Yat-sen University, Guangzhou, 510080 Guangdong China; 4 NHC Key Laboratory on Assisted Circulation of the First Affiliated Hospital, Sun Yat-sen University, Guangzhou, 510080 Guangdong China; 5 Department of Breast and Thyroid Surgery, The First Affiliated Hospital, Sun Yat-sen University, Guangzhou, 510080 Guangdong China; 6 Department of Breast Surgery, The First Affiliated Hospital of Guangzhou Medical University, Guangzhou, 510080 Guangdong China; 7 State Key Laboratory of Respiratory Diseases and Guangzhou Institute of Respiratory Diseases, The First Affiliated Hospital of Guangzhou Medical University, 151 Yanjiang Road, Guangzhou, 510000 Guangdong China

## Correction to: Cell Biosci (2019) 9: 44 10.1186/s13578-019-0310-2

In the publication of this article [1], there is an error in one of the contributing author names.

The error: ‘Yongbo Huan’

Should instead read: ‘Yongbo Huang’

This has now been updated in the original article [1].

## References

[1] Zhang L, Lian R, Zhao J, Feng X, Ye R, Pan L, Wu J, Li M, Huang Y, Cai J (2019). IGFBP7 inhibits cell proliferation by suppressing AKT activity and cell cycle progression in thyroid carcinoma. Cell Biosci.

