# Supplementary figures and images for: IGFBP7 inhibits cell proliferation by suppressing AKT activity and cell cycle progression in thyroid carcinoma
Source: Cell Biosci. 2019 Jun 6;9:44. doi: 10.1186/s13578-019-0310-2 (PMC6555742; doi:10.1186/s13578-019-0310-2)

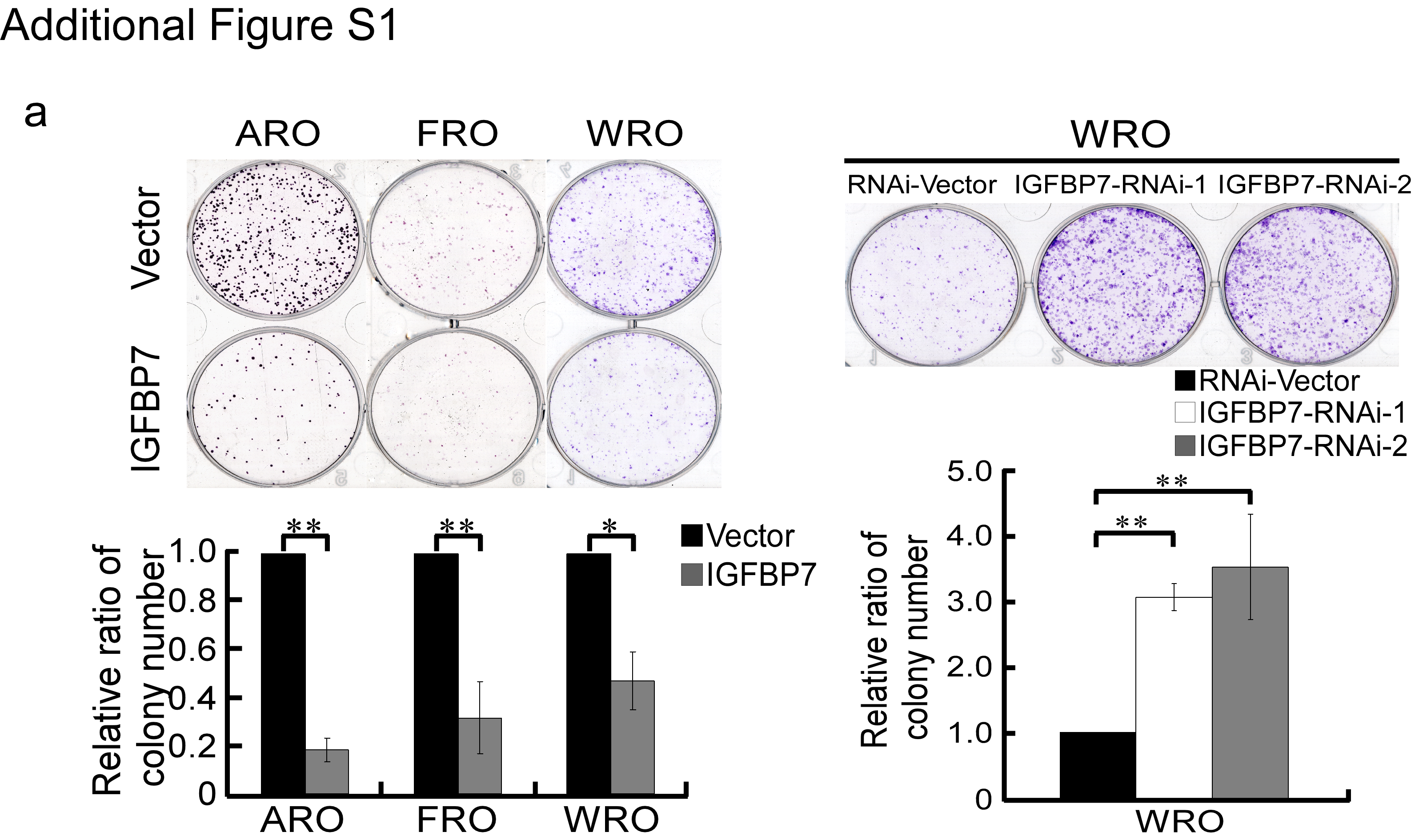

Supplement: Supplementary file 1 — Additional file 1: Figure S1. IGFBP7 inhibits proliferation of thyroid cancer cells in vitro. (a) Representative micrographs (upper) and relative quantification (lower) of the indicated cells as evaluated by the colony formation assay. Data represent the mean ± S.D. of three independent experiments. A two-tailed Student’s t-test was used for statistical analysis (*P < 0.05, **P < 0.01). [file 13578_2019_310_MOESM1_ESM.tif]
